# Association of aryl hydrocarbon receptor transactivating activity, a potential biomarker for persistent organic pollutants, with the risk of gestational diabetes mellitus

**DOI:** 10.1038/s41598-021-82794-0

**Published:** 2021-02-04

**Authors:** Sunmin Park, Suk Chon, So Young Park, Soojin Yun, Sei Hyun Baik, Jeong Taek Woo, Sang Youl Rhee, Youngmi Kim Pak, Sung-Hoon Kim

**Affiliations:** 1 Department Food and Nutrition, Hoseo University, Asan, Korea; 2 Department of Endocrinology and Metabolism, Kyung Hee University College of Medicine, Seoul, Korea; 3 Department of Endocrinology and Metabolism, Kyung Hee University Hospital, Seoul, Korea; 4 Department of Endocrinology and Metabolism, Korea University College of Medicine, Seoul, Korea; 5 Department of Physiology, Kyung Hee University College of Medicine, Seoul, Korea; 6 Department of Internal Medicine, Yonsei University Wonju College of Medicine, Wonju, Korea

**Keywords:** Endocrinology, Risk factors

## Abstract

Persistent organic pollutants(POPs) are suggested to be potential risk factors for gestational diabetes mellitus(GDM). We examined the hypothesis that the aryl hydrocarbon receptor trans-activating(AhRT) activity, a potential biomarker for the presence of POPs, could be a GDM risk factor in pregnant women. A total of 390 GDM and 100 normal pregnant(non-GDM) subjects in the Korea National Diabetes Program cohort voluntarily participated. We measured AhRT activity and concentrations of ATP and reactive oxygen in the serum collected at the screening of the participants for GDM using recombinant Hepa1c1c7 cells. Odds ratios(ORs) and 95% confidence intervals(CIs) were estimated using multivariable logistic regression models. The sensitivity and specificity of AhRT activity for GDM diagnostics were measured by receiver operating characteristic(ROC) analysis. Body mass index at pre-pregnancy and delivery and systolic blood pressure were significantly higher in the GDM group. AhRT activity was higher, and ATP concentrations were lower in the GDM group than the non-GDM group(*P* < 0.0001). AhRT activity was significantly higher in the GDM group(OR 29.3, 95% CI 10.9–79.1) compared with non-GDM(*P* < 0.0001). Serum glucose concentration at 1 h after a 50 g glucose challenge(glucose-50) was moderately correlated with AhRT activity(r^2^ = 0.387) and negatively correlated with ATP production(r^2^ =  −0.650). In the ROC curve, AhRT activity had 70.9% sensitivity and 90.0% specificity for glucose-50, a GDM screening method. In conclusion, this study suggests that serum AhRT activity is positively associated with the risk of GDM.

## Introduction

Gestational diabetes mellitus (GDM) typically develops at the end of the 2nd trimester (24–28 weeks) of pregnancy when insulin secretion cannot compensate for increased insulin resistance. GDM is characterized by the disturbance of glucose metabolism^1,2^. The global prevalence of GDM varies between 2% and more than 18%. There is an ethnic difference: Asians have higher GDM than Caucasians^3,4^. In GDM, the pregnant mother experiences a sudden increase in insulin resistance induced by pregnancy, and it also predicts an elevated risk of developing type 2 diabetes later in life^2^.

Risk factors for GDM include advanced maternal age, high body mass index (BMI) at pre-pregnancy, greater weight gain during pregnancy than the recommendation, and family history of diabetes^5^. The global increase in GDM prevalence may also be associated with unknown environmental factors. One potential factor is the increased exposure to endocrine-disrupting chemicals (EDCs). Accumulating evidence has demonstrated that exposure to EDCs is linked to impaired glucose metabolism and type 2 diabetes as shown in cross-sectional and prospective studies in humans and experimental in vivo and in vitro studies^6^. Among EDCs, the exposure to dichlorodiphenyldichloroethylene, a metabolite of the pesticide dichlorodiphenyltrichloroethane, exhibits a moderate relationship with diabetes development in human studies^6^. Exposure of polychlorinated biphenyls (PCBs), bisphenol A, phthalates, perfluorinated and poly-fluoroalkyl substances (PFAS), polybrominated diphenyl ethers (PBDE) have little research to support their associations with diabetes risk^6^. Some EDCs are considered persistent organic pollutants (POPs)^7^. POPs are lipophilic chemicals with long half-lives due to resistance to environmental degradation processes. POPs that are incorporated into the food chain accumulate in the adipocytes of humans over time^8^. Mitochondrial dysfunction due to POPs subsequently contributes to increased insulin resistance^9^. Therefore, POPs can be potential risk factors for obesity, type 2 diabetes, and GDM^7,8^. However, an optimal index for exposure to POPs has not been established. Most studies have used plasma concentrations of 76 POPs as an index, which is determined by standard procedures using gas- and liquid chromatography (LC) with mass spectrophotometry^10^. Limited numbers of POPs have been identified due to the limited number of facilities available to measure them, the large volume of blood needed, and the high cost. Most POPs bind to aryl hydrocarbon receptors with high affinity and they enter into the cells and bind to dioxin-response elements of their promotors that contribute to transcriptional activation of multiple genes, including cytochrome p450 1A1 (*CYP1A1*)^11^. POPs act as potent activators of aryl hydrocarbon receptor transactivating (AhRT) and AhRT bioactivity can be an index of the POPs levels, including dioxin and dioxin-like substances^9^. However, AhRT is also activated by endogenous indole derivatives such as kynurenine produced by activation of the tryptophan–kynurenine pathway^12^. Under diabetic conditions, the tryptophan-kynurenine pathway stimulates the increase of endogenous indole derivatives^12,13^. A highly sensitive cell-based AhR dependent luciferase activity assay has been developed to determine AhRT activity. Serum dioxin levels measured by AhRT activity exhibit a strong correlation with its levels measured by Gas/Liquid chromatography-mass spectrophotometry^9^. A positive correlation between dioxin and AhRT activity has been shown in the Swedish elderly population, and AhRT activity had a negative association with ATP production in the mitochondria^14^. Patients with diabetic nephropathy also have increased serum AhRT activity^15^. Thus, the impact of endogenous indole derivatives may be minimal due to working as weak agonists.

A recent study has demonstrated that serum concentrations of POPs, including mainly chlorinated PCBs and some PFAS and PBDE, have positive associations with GDM risk in US non-obese women in the multi-center prospective cohort study^10^ and pregnant Greek women^11^. Here, we examined the hypothesis that AhRT bioactivity in the serum is positively associated with GDM risk in Korean pregnant women at 24–28 weeks of pregnancy. AhRT activity and ATP production in the serum can be biomarkers for the POPs exposure.

## Results

### Characteristics of GDM and non-GDM participants

At pre-pregnancy, age was not significantly different between the GDM and non-GDM groups. However, height was higher in the non-GDM group than the GDM group (*P* < 0.001), whereas body weight and BMI were much higher in the GDM group (58.6 ± 10.1 and 23.0 ± 3.8) than the non-GDM group (55.0 ± 5.7 and 20.7 ± 1.9, *P* < 0.001; Table 1).

**Table 1:** Characteristics of pregnant women with gestational diabetes (GDM) and normal glucose tolerance (Non-GDM).

	GDM (n = 390)	Non-GDM (n = 100)	*P* value
**Before pregnancy demographics**			
Age (years)	34.1 ± 3.5	34.2 ± 3.3	0.94
Height (cm)	159.5 ± 4.9	163.1 ± 5.0	< 0.001
Body weight (kg)	58.6 ± 10.1	55.0 ± 5.7	< 0.001
Body mass index (kg/m^2^)	23.0 ± 3.8	20.7 ± 1.9	< 0.001
**At 24–28 week of the pregnancy for GDM screening**			
1 h glucose after 50 g OGTT (mg/dL)	166.6 ± 23.2	112.8 ± 15.8	< 0.001
Systolic blood pressure (mmHg)	110.1 ± 10.8	104.3 ± 13.4	< 0.001
Diastolic blood pressure (mmHg)	67.7 ± 8.01	64.4 ± 16.5	0.061
**At delivery demographics**			
Body weight (kg)	68.2 ± 9.8	69.5 ± 6.3	0.218
Body mass index (kg/m^2^)	26.6 ± 4.2	22.5 ± 9.0	0.002
Pregnancy duration (weeks)	38.8 ± 1.5	39.4 ± 1.2	< 0.001
Baby Apgar score 1 min	8.2 ± 0.8	8.2 ± 0.8	0.72
Baby Apgar score 5 min	9.0 ± 0.6	9.0 ± 0.6	0.38
Baby body weight at birth (g)	3285 ± 472	3287 ± 417	0.967

Apgar, appearance, pulse, grimace, activity, and respiration.

At weeks 24–28 of pregnancy screening for GDM, serum glucose concentrations were higher in the GDM group (166.6 ± 23.2 mg/dl) than the non-GDM group (112.8 ± 15.8 mg/dl) at 1 h after 50 g glucose challenge (*P* < 0.001; Table 1). Systolic blood pressure (SBP) was higher in the GDM group than the non-GDM group (*P* < 0.001), whereas DBP was not significantly different between the GDM and non-GDM groups. After GDM diagnosis, serum glucose concentrations were maintained in a normal range in pregnant women with GDM by medication and diet therapy. BMI at delivery was higher in the GDM group than the non-GDM group (*P* = 0.002), although body weight gain was much lower in the GDM group (9.6 kg) than the non-GDM group (14.5 kg; Table 1). Body-weight increased within the recommendations in both GDM and non-GDM groups. The pregnancy duration was shorter in the GDM group than the non-GDM group (Table 1). The birth weights of the infants were not significantly different between the two groups. Apgar at 1 and 5 min were not significantly different between the two groups (Table 1). The results indicated that pregnancy outcomes were not significantly different.

### AhRT activity and ATP contents in non-GDM and GDM women

**Figure 1:**
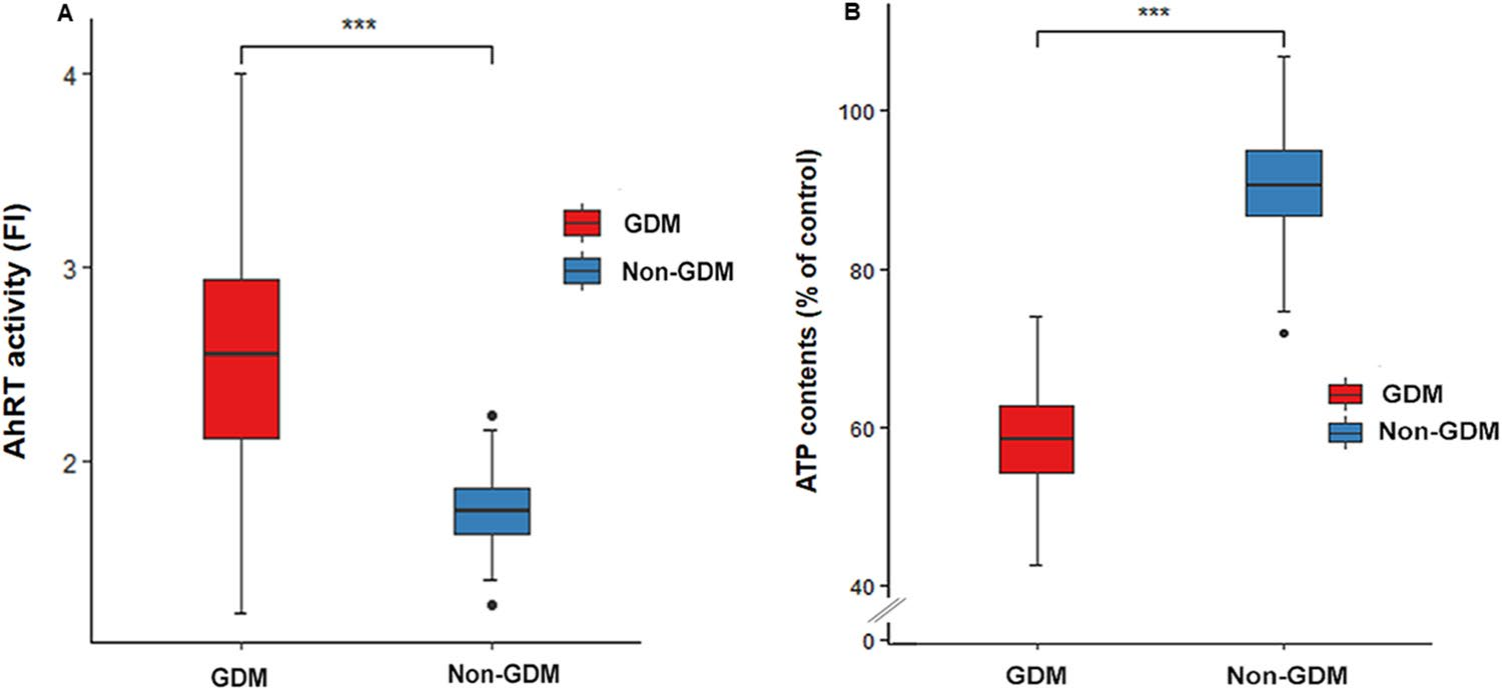
Aryl hydrocarbon receptor transactivating (AhRT) bioactivity and ATP contents of the serum in the non-GDM and GDM groups. (**A**) AhRT bioactivity. AhRT activity was measured by the modified CALA assay using the serum from 390 GDM and 100 non-GDM participants. AhRT activity was presented as fold induction (FI) over the AhRT of the 10% CS-HS-treated control cells. (**B**) ATP content. ATP concentration of 10% sample serum-treated cells from the participants was measured by a CellTiter-Glo luciferase kit. It was calculated as % of ATP content in control cells treated with 10% CS-HS-treated control cells. ***Significantly different from the GDM group at *P* < 0.0001.

AhRT activity was significantly higher in GDM than non-GDM women (*P* < 0.0001; Fig. 1A), and ATP concentrations were significantly lower in GDM than the non-GDM women (*P* < 0.0001; Fig. 1B).

**Figure 2:**
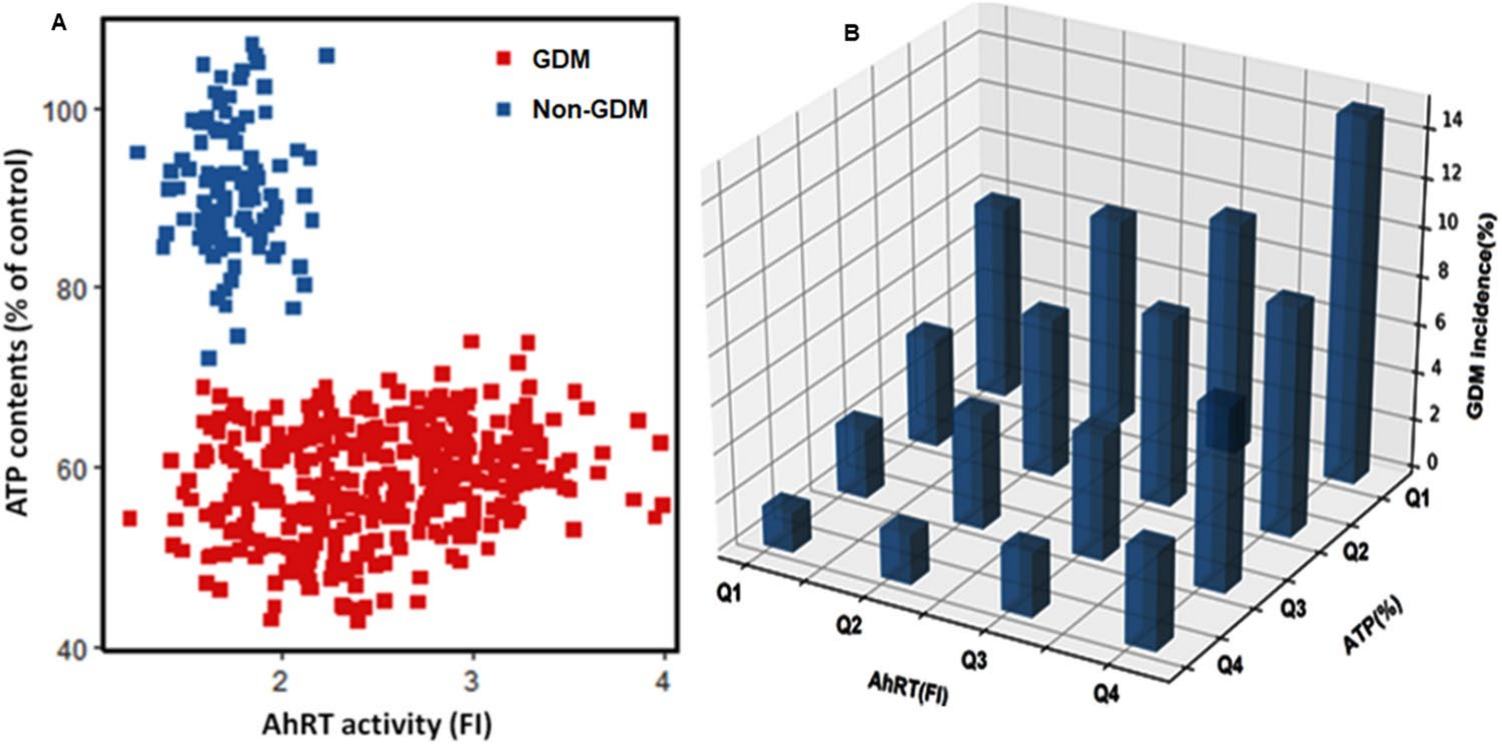
The correlation of serum glucose concentration at 1 h after 50 g glucose challenge, aryl hydrocarbon receptor transactivating (AhRT) bioactivity, and ATP concentration. (**A**) Relationship between AhRT bioactivity (FI) and ATP contents (%). (**B**) The proportion of GDM (%), according to the quartiles of AhRT activity (FI) and ATP concentration (%). GDM proportion was calculated by the number of women with GDM divided by the total number of women in each quartile. AhRT bioactivity was measured by the modified CALA assay using the serum from 390 GDM and 100 non-GDM participants. AhRT activity was presented as fold induction (FI) over the AhRT of the 10% CS-HS-treated control cells. ATP concentration of 10% sample serum-treated cells was measured by a CellTiter-Glo luciferase kit. It was calculated as % of ATP content in control cells treated with 10% CS-HS-treated control cells.

Figure 2A shows that non-GDM women had ≤ 2.0 AhRT activity, but about 35% of GDM women also had ≤ 2.0 AhRT activity, although GDM women had higher serum glucose concentrations. However, about 65% of GDM women had much higher AhRT activity (Fig. 2A). Interestingly, ATP contents exhibited a clear separation by GDM: most women with non-GDM included ≥ 80% of the control, but GDM women comprised ≤ 75% of the control group (non-GDM women) in ATP contents (Fig. 2A). In clustering analysis of AhRT activity and ATP contents, GDM and non-GDM women showed a clear separation (Fig. 2A). These results indicated that mitochondrial dysfunction with increased AhRT contents might be involved in GDM risk.

When the risk of GDM was analyzed according to the quartiles of AhRT activity or ATP concentrations, most subjects in the upper fourth quartile (Q4) of AhRL (≥ 2.85 FI) and the lower first quartile (Q1) of ATP contents had GDM (Fig. 2B). In contrast, few subjects in the lowest quartile (Q1) of AhRT (< 1.85 FI) and the highest quartile (Q4) of ATP contents (67.1%) had GDM. These results indicate that AhRT activity and ATP contents are the independent parameters for GDM development.

### Correlation of AhRT activity, and ATP and reactive oxygen species (ROS) concentrations with GDM-related clinical parameters

AhRT activity was positively correlated with serum glucose concentrations at 1 h after a 50 g challenge and HbA1c (r^2^ = 0.387 and 0.219, respectively; Table 2). AhRT activity had a strong negative association with ATP contents and a positive association with ROS concentrations (r^2^ =  − 0.400) in GDM women (Table 2). AhRT activity had a weak positive correlation with age, BMI at pre-pregnancy and delivery, SBP, and DBP (Table 2). In contrast to AhRT activity, ATP contents were strongly and negatively correlated with plasma glucose at 1 h after 50 g glucose challenge and weakly negatively associated with SBP and DBP (Table 2). AhRT activity had a positive association with HOMA-IR, an index of insulin resistance, but not with HOMA-B, an indicator of insulin secretion (Table 2). HbA1c contents also exhibited a positive association with AhRT activity and ROS concentrations. ROS concentrations had a weak positive correlation with age and baby Apgar score at 5 min.

**Table 2:** Pearson correlations coefficients(r) between the aryl hydrocarbon receptor transactivating (AhRT) activity and metabolic parameters in the pregnant women with gestational diabetes.

	OGTT after 50 g glucose challenge	AhRT activity	ATP contents	ROS concentrations
AhRT activity	0.387***	1.0	− 0.400***	0.213***
ATP contents	− 0.650***	− 0.400***	1.0	− 0.086
ROS concentrations	− 0.032	0.181***	− 0.086	1.0
Age	0.00007	0.118*	− 0.011	0.175***
BMI at prepregnancy	0.181**	0.113*	− 0.003	0.071
BMI delivery	0.144**	0.156**	− 0.049	0.065
HbA1c	0.507***	0.219***	− 0.030	0.187**
0 h glucose at 50 g OGTT	0.359***	0.135**	0.051	− 0.003
1 h glucose at 50 g OGTT	1.0	0.387***	− 0.650***	− 0.032
0 h insulin at 100 g OGTT	0.077	0.128*	0.016	0.015
1 h insulin at 100 g OGTT	− 0.163**	0.050	0.098	0.044
HOMA-IR	0.167***	0.145**	0.028	0.017
HOMA-B	− 0.026	0.095	− 0.020	0.010
Systolic blood pressure	0.208***	0.077	− 0.164***	− 0.013
Diastolic blood pressure	0.158***	0.145**	− 0.131***	− 0.021
Weight difference	− 0.163**	0.032	0.064	0.006
Baby body weight at birth	− 0.0010	0.040	− 0.035	0.093
Baby Apgar at 1 min	− 0.034	0.060	− 0.005	0.036
Baby Apgar at 5 min	− 0.007	0.093	− 0.057	0.110*
After delivery BMI	0.132*	0.1553**	0.053	0.055

*ROS* reactive oxygen species, *BMI* body mass index, *OGTT* oral glucose tolerance test, *HOMA-IR* homeostatic model assessment for insulin resistance, *HOMA-B* HOMA for β-cell function, *Apgar* appearance, pulse, grimace, activity, and respiration.

*Significantly correlated at *P* < 0.05, ** *P* < 0.01, and *** *P* < 0.001.

### Association of age, BMI, and AhRT activity with GDM risk

Age was not associated with the risk of GDM in logistic regression analysis (Table 3). BMI at pre-pregnancy and delivery was highly associated with GDM risk by 1.316 and 3.615-fold (*P* < 0.0001). Interestingly, AhRT activity was strongly associated with GDM risk by 29.3-fold (95% CI 10.88–79.05, *P* < 0.0001; Table 3). Therefore, AhRT bioactivity could be used as a diagnostic index and potential risk factor. However, serum glucose concentration at 1 h after 50 g glucose challenge was a better index than AhRT (the difference of their estimate = 0.1468, 90% CI 0.1119–0.1817, *P* < 0.0001; Table 3).

**Table 3:** Multiple logistic regression analysis of gestational diabetes mellitus.

	β	SE	OR	95% CI	*P*
Age	0.125	0.169	1.283	0.661–2.49	0.4611
BMI at prepregnancy	0.275	0.060	1.316	1.169–1.481	< 0.0001
BMI at delivery	0.643	0.119	3.615	2.264–5.772	< 0.0001
AhRT activity	1.690	0.036	29.33	10.88–79.05	< 0.0001

The cutoffs are following: 35 years old for age; 22.0 and 25.0 kg/m^2^ for body mass index (BMI) at prepregnancy and at delivery, respectively; and 2.0-fold induction for aryl hydrocarbon receptor transactivating (AhRT) activity.

Adjusted for age, blood glucose at OGTT 50 g, HOMA-IR, systolic blood pressure, diastolic blood pressure, and BMI at prepregnancy and at delivery.

### Sensitivity and specificity of AhRT bioactivity to GDM risk in receiver operating characteristic (ROC) plot

**Figure 3:**
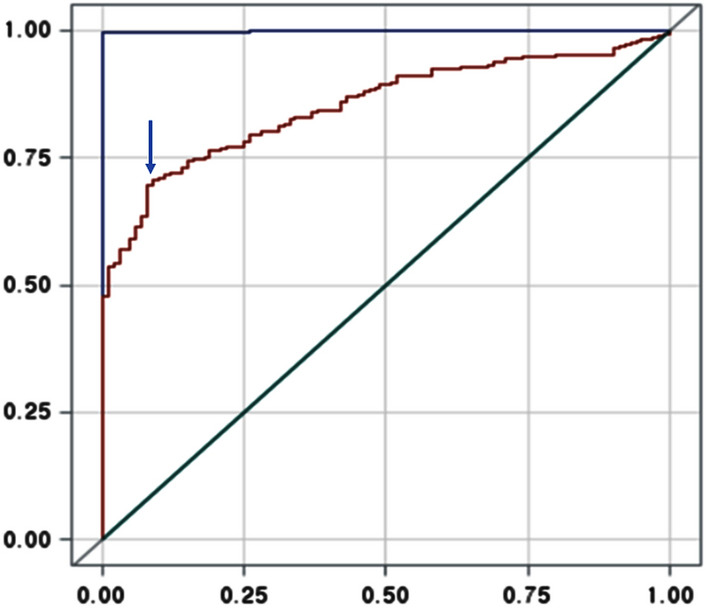
Receiver operating characteristic (ROC) curve for AhRT activity in 390 GDM and 100 non-GDM participants. The ROC curve determined the sensitivity and specificity of AhRT activity for GDM risk. The top and bottom lines in the ROC curves corresponded to GDM predictive tests. The top line represented serum glucose concentration from a 50 g glucose challenge for GDM prediction, and its area under the curve (AUC) was 0.9993. The bottom line indicated AhRT bioactivity to GDM prediction, and its AUC was 0.8527. These results indicated the AhRT bioactivity could be considered as a GDM risk factor. ROC2 was the reference.

The ROC curve showed that AhRT had 70.9% sensitivity and 90.0% specificity with a 1.979 cutoff value of AhRT for GDM diagnosis (Fig. 3). Area of AhRT and serum glucose concentrations and serum glucose concentrations at 1 h after 50 g glucose challenge was 0.8527 (95% CI 0.8173–0.8873) and 0.9993 (0.9976––1.0000), respectively (Fig. 3). The 95% CI for both parameters did not include the reference value of 0.5. These results indicated that AhRT activity is representative of GDM risk and can be used to assess for GDM risk.

## Discussion

POPs are reported to be a risk factor for type 2 diabetes and diabetic nephropathy^7^. AhRT bioactivity is known to be negatively associated with pancreatic β-cell function, and it is a biomarker of exposure to POPs^16^. AhRT activity can also be a risk factor for GDM^16^. It is difficult to directly measure POPs in the blood since POPs contain various components. AhRT bioactivity can be an index of POP levels, including dioxin and dioxin-like substances^17^. In this study, we investigated whether POPs could be risk factors for GDM in Korea by measuring AhRT activity as a potential biomarker of POPs in samples from pregnant women. To the best of our knowledge, the present study is novel research and the first to demonstrate the association of AhRT bioactivity with GDM risk in pregnant women.

GDM develops during the 3rd trimester of pregnancy when elevated insulin resistance in the mother cannot be overcome by compensatory insulin secretion from pancreatic β-cells^1^. Although the cause of insulin resistance in GDM is different from type 2 diabetes, the etiologies of GDM and type 2 diabetes are similar^1^. The factors linked to increased insulin resistance in type 2 diabetes are also the primary risk factors for GDM, including weight gain. However, the high incidence of GDM in Asians is related to low insulin secretion capacity, and the inability to compensate for insulin resistance^1,18^. GDM consequences include short- and long-term maternal and child health problems. GDM increases the risk of preterm birth, preeclampsia, stillbirth, and macrosomia during pregnancy. Women with GDM have an increased risk of subsequent obesity, type 2 diabetes, and cardiovascular diseases, and their children are at risk of childhood obesity^2,19^. It is important to avoid the modifiable risk factors for GDM, including pre-pregnancy obesity and excessive weight gain before and during pregnancy^2^. Advanced maternal age is a major unmodifiable risk factor for GDM. However, it is better to investigate modifiable risk factors to prevent GDM.

Exposure to POPs is one of the modifiable risk factors for GDM. Accumulating evidence from experimental studies suggests that pancreatic β-cells can be considered a sensitive target of dioxin toxicity, which reduces β-cell function, thereby increasing the risk of type 2 diabetes and GDM^20^. Environmental pollution has been markedly increasing in Asian countries over the last 3 decades, and Asians already have lower insulin secretion capacity. These two factors may influence the increase in type 2 diabetes and GDM risk. Exposure to EDCs is one of the environmental and modifiable, but unavoidable risk factors^21^. The present study showed that AhRT activity was much higher in the GDM group than in the non-GDM group. The sensitivity and specificity of AhRT activity for GDM were 0.8523 and 0.9991, respectively, indicating that AhRT activity could be an independent risk factor for GDM. Therefore, decreasing the accumulation of POPs may be a useful strategy for reducing GDM risk.

The blood is the only sample often used for measuring exposure to POPs. However, serum POPs concentrations may not be a well-representative index of POPs exposure since POPs accumulate in the fat tissues^21^. Body fat contents may also affect the serum concentrations of POPs. As POPs are composed of various chemicals, it is difficult to measure all POPs in the blood due to the many different types of chemicals comprising POPs, the limited number of available standard compounds for gas- and liquid chromatography/mass spectroscopy, the sensitivity of the equipment, and required amounts of samples. Therefore, another method is necessary for measuring POP exposure. Serum AhRT bioactivity can be an index of serum POP concentrations, including dioxin and dioxin-like substances. Most POPs bind to AHR with a high affinity to activate dioxin-response elements of the promoters, contributing to transcriptional activation of various genes that influence the risk of metabolic diseases, including type 2 diabetes.

EDCs contain some POPs (e.g., PFAS) and non-POPs (i.e., phenols, phthalates, and parabens), and they are reported to be associated with dysregulated glucose metabolism in GDM and type 2 diabetes^22,23^. Many epidemiological studies have demonstrated an association between POP exposure, metabolic syndrome, and type 2 diabetes^7,24^. However, the effects of POPs on GDM need more studies to be conclusive^25^. POPs are lipophilic and hydrophobic pollutants that are stored in fatty tissues. Since POPs are resistant to metabolic degradation, they tend to accumulate in humans^26^. Previous studies have demonstrated that POPs, including PCBs, are involved in the development of diabetes mellitus (DM) type 2 and insulin resistance^27,28^. The present study revealed that AhRT bioactivity in the serum is a reliable indicator of POP exposure, and it is correlated with GDM and insulin resistance, but not insulin secretion. EDCs, including POPs, are reported to increase AhRT bioactivity and ROS production and induce mitochondrial dysfunction, which exacerbates metabolic syndrome^29^. The present study suggests that the activation of serum AhRT bioactivity, as with POP accumulation, contributes to GDM risk by reducing ATP production and increasing ROS production in the mitochondria that are subsequently related to increased insulin resistance.

This study had some limitations. First of all, POPs are not the only AhRT agonists; the endogenous indole derivatives, including tryptophan metabolites, from microbiota production and dietary components, contribute to influencing AhRT activity^30^. The interaction of endogenous indole derivatives within the ligand-binding domain of AhRT appears to differ from that observed with high-affinity ligands such as dioxin. Therefore, the serum concentrations of endogenous indole derivatives may contribute to a basal level in the participants, although circulating amino acid concentrations are elevated^31^, and the tryptophan-kynurenine pathway is activated in diabetic patients, including GDM^12,13^. Since the endogenous indole derivatives have a much lower affinity to AhRT than POPs^30^, the AhRT activation measured in the present study is more likely to be associated with POP exposure. Second, GDM development is involved in socioeconomic factors and lifestyle factors, including nutrient intake and physical activity^32,33^. These factors should have used as adjusting covariates, but they were not measured in the present study. Age, BMI, and serum glucose concentrations at 50 g glucose challenge were used as covariates in the present study.

## Conclusion

This study suggested that serum AhRT activity has a positive association with GDM risk in Korean women and it is a reliable potential biomarker for exposure to POPs. Exposure to POPs might be a potential risk factor for the development of GDM in pregnant women in Korea and possibly other countries. Further research is needed to evaluate the role of AhRT activation in GDM development.

## Methods

### Participants

The Korea National Diabetes Program (KNDP) cohort consists of Koreans who have or who at high risk of developing type 2 diabetes who have enrolled at 13 hospitals nationwide since 2006 and were regularly followed up during the 9 years. Among the participants in the KNDP GDM cohort, pregnant women were diagnosed by a universal two-step GDM screening program at 24–28 weeks of gestation. The first step was a 50-g glucose challenge test, and women with a positive result underwent a 3-h 100-g oral glucose tolerance test (OGTT). This study was conducted with the 390 women with GDM among the KNDP cohort whose clinical data and blood samples at 24–28 weeks of gestation were available for AhRT assay and data analysis. Among the pregnant women who were tested for GDM screening, 100 non-GDM women volunteered to participate in the control group. All procedures of the KNDP GDM cohort study were conducted following the guidelines and regulations of the Declaration of Helsinki revised in 2004. This study was approved by the Institutional Review Board (IRB) of Kyung Hee University Hospital (IRB No. KHUH 2015–04-201–016). Informed consent was obtained from all participating subjects.

### Laboratory measurements and physiological indexes

Plasma glucose concentrations were measured by the glucose oxidase method using a YSI 2300 STAT (YSI; Yellow Springs, Ohio). Plasma insulin concentrations were measured using a human-specific radioimmunoassay kit (Linco Research, St. Charles, MO). Homeostatic model assessments for insulin resistance (HOMA-IR) and β-cell function (HOMA-B) were calculated using the equations as previously reported^34^. HbA_1c_ levels were determined at the initial diagnosis of GDM and before delivery by the Variant II HbA1c program (BioRad; Hercules, CA). Gestational hypertension was diagnosed if blood pressure was ≥ 140 for systolic (SBP) and 90 mmHg for diastolic (DBP).

### Pregnancy outcomes

Preterm delivery was defined as delivery before 37 weeks of gestation, whereas macrosomia was considered a birth weight of 4000 g or greater^1,33^. Large for gestational age was defined as a birth weight ≥ 90th percentile based on a Korean reference population. The Apgar score was defined as low if a score of < 7 was recorded 5 min after delivery.

### AhRT bioactivity assay

The modified cell-based AhR ligand activity assay was conducted to quantify the dioxin response element (DRE)-dependent luciferase activity (modified CALA assay)^9,35,36^. Hepa1c1c7, Mouse Hepatoma cells, were cultured in α-minimum essential medium supplemented with 10% fetal bovine serum and 1% penicillin/streptomycin at 37 °C in a 5% CO_2_ atmosphere. Cells seeded at 1 × 10^5^/well in a 6-well plate. The cells were co-transfected with the pGL4-DRE-Luc (puromycin +) and pRL-mTK Renilla reporter plasmids using Superfect (Qiagen, Valencia, CA, USA). Puromycin (1 μg/ml)-resistant stable colonies were selected after a 3-week incubation. The clones, containing 2,3,7,8-tetrachlorodibenzo-p-dioxin (TCDD; AhR agonist)-dose-dependent activity, were selected^15^. The stable cells with a 70% confluent state were treated with heat-inactivated human serum samples or a charcoal-stripped human serum (control serum) in Dulbecco’s modified Eagle medium for 24 h. Luciferase activities were measured using a Dual-Glo Luciferase assay system (Promega, Madison, WI, USA) in a luminometer (Berthold, Bad Wildbad, Germany). The luciferase activity was subsequently normalized against *Renilla* luciferase activity. AhRT bioactivities of sample serum-treated cells were reported as fold induction over those of the control serum-treated control cells. Standard curves were prepared using luciferase activities (AhRT bioactivity) of the cells exposed to serially diluted TCDD (0–50 pM) for 24 h in the presence of a control serum. When the AhRT bioactivity in a serum sample was converted to TCDD equivalents (pM) using the standard curve (0–10 pM TCDD), a 0.1-fold induction of AhRT bioactivity was equivalent to 0.37 pM TCDD equivalent.

### ATP content assay in the serum of pregnant women

The mitochondrial inhibition induced by serum samples was evaluated by measuring intracellular ATP contents^9,37^. Hepa1c1c7 cells were co-transfected with the pRL-mTK *Renilla* and pcDNA3.1 (neomycin +) plasmids using Superfect (Qiagen, Valencia, CA, USA). G418-resistant stable colonies were selected for 3 weeks, and then the clones showing stable *Renilla* luciferase activity were selected. Stable clones (5 × 10^4^/well) were treated with serum samples of pregnant women (10 ul) or control serum in 96-well plates for 48 h. The intracellular ATP contents of the serum-treated cells were measured through the luciferin-luciferase reaction using CellTiter-Glo luciferase kits (Promega, Madison, WI, USA). *Renilla* luciferase activity was determined by adding an equal amount of Stop & Glo substrate solution from the Dual-Glo Luciferase assay kit (Promega, Madison, WI, USA). ATP concentrations were normalized to *Renilla* luciferase activity. The dose-dependent effects of four PCBs and TCDD on ATP concentration were also determined in the presence of control serum. The results were presented as % control of ATP content in control cells treated with control serum. Both intra- and inter-assay coefficients of variation were less than 6.0%.

### Intracellular ROS assay of the serum of pregnant women

Hepa1c1c7 cells (5X10^4^/well) in a 96-well plate were cultured for 24 h in DMEM containing 10% FBS. After media was switched to 90 μl phenol red-free DMEM, cells were treated for 48 h with 10 μl heat-inactivated human serum samples or charcoal-stripped human serum (CS-hS, control) to obtain a final concentration of 10% serum. We measured ROS using 5-(and-6)-chloromethyl-2′,7′-dichlorodihydrofluorescein diacetate, acetyl ester (CM-H2DCFDA, Molecular Probes). ROS intensities were normalized by Hoechst nuclei staining to eliminate the well-to-well seeding error as described^9^.

### Statistical analysis

Statistical analyses were performed using SAS, version 9.1, and data are expressed as means ± standard deviation. The sample size was calculated using the G-power program (α = 0.05, power = 0.95). The effect size was assigned to 0.15 since the standard deviation of blood glucose concentrations in Korean pregnant women was about 15–20%. The sampling ratio between GDM and non-GDM groups was about 0.2. Considering these factors, the total number of samples was about 230, and the number of participants in the present study (GDM: 390 and Non-GDM: 100) was sufficient. The statistical differences between the non-GDM and GDM groups were determined using a two-tailed sample t-test for continuous variables and χ^2^ test for categorical variables. The association between GDM and AhRT activity was evaluated with multivariate logistic regression analyses after adjusting for age and BMI at pre-pregnancy and 24–28 weeks. Adjusted ORs (95% CI) for the GDM risk to pregnancy outcomes were calculated using a logistic analysis. The correlations between AhRT activity and pregnancy outcomes were analyzed with Pearson correlation coefficients. The ROC curve was measured to determine the sensitivity and specificity of AhRT activity for GDM risk. A value of *P* < 0.05 was taken to indicate statistical significance.
